# Genetic Characterization of the Factor VIII Gene in a Cohort of Colombian Patients with Severe Hemophilia A with Inhibitors

**DOI:** 10.3390/hematolrep14020022

**Published:** 2022-05-04

**Authors:** Samuel Sarmiento Doncel, Gina Alejandra Diaz Mosquera, Ronald Guillermo Pelaez, Javier Mauricio Cortes, Carol Agudelo Rico, Francisco Javier Meza Cadavid, Nelson Ramirez Plazas, Ivan Alfredo Perdomo Amar, Jorge Enrique Peña Siado, Fabian Andres Parrado Rey, Cesar Alberto Montaño, Alexys Maza Villadiego

**Affiliations:** 1 Integral Solutions SD SAS, Integral Solutions Research, Bogota 110121, Colombia; gdiaz@integralsolutionssd.com (G.A.D.M.); jmauriciocortes@gmail.com (J.M.C.); carolagudelor@gmail.com (C.A.R.); mezafco@gmail.com (F.J.M.C.); nelsonramirez59@yahoo.com (N.R.P.); ivapera0001@hotmail.com (I.A.P.A.); jpenasiado@gmail.com (J.E.P.S.); andres.parrado.md@gmail.com (F.A.P.R.); camm@utp.edu.co (C.A.M.); almazz02@yahoo.es (A.M.V.); 2 Life Sciences and Health Research Group, Graduates School, CES University, Medellin 050021, Colombia; rpelaez@ces.edu.co; 3 Hospital Universitario San Jorge, Pereira 660002, Colombia; 4 Hospital Universitario Hernando Moncaleano Perdomo de Neiva, Neiva 410010, Colombia

**Keywords:** factor VIII, hemophilia A, inhibitor, mutation, X chromosome

## Abstract

Hemophilia A is an X-linked bleeding disorder caused by mutations in the FVIII gene. Genetic factors have been shown to be a risk factor for the development of inhibitors. We aimed to identify the specific variations of the FVIII gene of patients with hemophilia A with inhibitors and their association with the inhibitor titer. Methods: Cross-sectional descriptive study. We included 12 Colombian patients from a health care provider, “Integral Solutions SD”, who underwent analysis of genetic material (DNA), which was reported by the Molecular Hemostasis Laboratory in Bonn, Germany. Results: All of these patients were diagnosed with severe hemophilia A with inhibitors; ages ranged between 6 and 48 years, with a median age of 13.5 years. Molecular analysis showed the inversion of intron 22 in six patients (50.0%), a small duplication in two patients (16.7%), the inversion of intron 1 in one patient (8.3%), a large deletion (8.3%), a nonsense mutation (8.3%) and a splice-site (8.3%), findings similar to those of other studies. A total of 58.3% of the patients presented inversion mutations with a high risk of developing inhibitors A total of 83.3% of the evaluated patients presented null mutations; however the presence of high inhibitor titers was 66.7%. The most frequent mutation was the inversion intron 22. Knowing the type of mutation and its association as a risk factor for generating inhibitors invites us to delve into other outcomes such as residual values of coagulation FVIII as well as its impact on the half-life of the exogenous factor applied in prophylaxis.

## 1. Introduction

Hemophilia A is a genetic disease linked to chromosome X characterized by the deficiency or absence of clotting factor VIII [1,2]. The Factor VIII gene has 26 exons distributed in 187,000 base pairs (bp), which encodes a polypeptide chain of 2351 amino acids (aa). Among the FVIII introns, intron 22 (32Kb) stands out for its large size. The most frequent mutations are inversion of introns 22 and 1, with 45% of cases of severe hemophilia A [3,4].

We can find several types of mutations in patients with hemophilia A, including small and large deletions, insertions or point mutations associated with deleterious changes such as missense mutation, nonsense mutations and splicing alterations [3,4].

The main complication of replacement therapy is the development of FVIII neutralizing inhibitors. Genetic factors are a risk factor for the development of inhibitors; in fact, between 20% and 30% of patients with severe hemophilia A develop this type of antibody [3,5,6].

Mutations in the absent or truncated FVIII protein are associated with a 20–80% risk of inhibitor development [6]. In mild or moderate hemophilia, nonsense mutations are associated with a 5% frequency of developing inhibitors [6]. Inversion of intron 22 (Inv22) causes 42% of severe hemophilia [3,7] and Inversion 1, 3% [3].

The development of inhibitors in patients with hemophilia A is multifactorial; these non-modifiable risk factors include the severity of hemophilia (the risk of inhibitors is 25–30% in severe hemophilia and 5% in mild or moderate hemophilia) [3,6,8]; family history of inhibitors [3]; and the genetic makeup of the patient. There are mutations associated with the absence of a product gene (null mutation), which confers a higher risk of generating inhibitors, e.g., inversion of introns 1 and 22, large deletions, nonsense mutations, and splice site mutations [3,6,9,10]. Missense or splicing mutations are considered low-risk mutations with an average frequency of developing inhibitors of less than 5% [3,9,10]. Inhibitor development significantly increases morbidity in this population and decreases their quality of life.

For this reason, the objective of this study was to identify the specific variations of the FVIII genes of patients with hemophilia A with inhibitors and their association with the inhibitor titer.

## 2. Methods

A cross-sectional descriptive study was conducted, which included Colombian patients of a health care institution in Colombia (Integral Solutions SD) with severe hemophilia A with inhibitors, and results of the genetic mutations were reported by the Laboratory of Molecular Hemostasis in Bonn, Germany. The information was collected through Integral Solutions SD medical records and delivered in an anonymous database to Integral Solutions Research.

Patients were eligible for the study if they met the inclusion criteria: diagnosis of severe hemophilia A (FVIII: <1%) with inhibitors (In Colombia for the year 2019, according to the high-cost account, 1.916 cases of hemophilia A were reported, of which 242 patients developed inhibitors), resulting in genetic sequencing and complete medical records. The exclusion criteria were acquired hemophilia A and female patients, so the study sample size was reached.

The descriptive analysis of the qualitative variables (absolute frequencies and percentages) and quantitative variables (position measurements) was performed, as well as the bivariate analysis, to determine the association between the type of mutation and the titer of the inhibitors. For this, we used the statistical software SPSS version 26, licensed by Integral Solutions Research.

Given that clinical histories were used, the following biases were considered: Information that was controlled by corroborating the data found in the clinical history against what was reported; selection bias, where the reference population was adequately established with the inclusion criteria, ensuring that the data of the variables are valid.

Integral Solutions SD provided the data anonymously to Integral Solutions Research and the confidentiality of the data and compliance with the standards of good clinical practice were guaranteed at all times.

## 3. Results

We analyzed data from 12 patients diagnosed with severe hemophilia A with inhibitors; the age range was 6 years to 48 years, with a median of 13.5 years. Of the 12 patients, 50% received immune tolerance induction (ITI), 16.7% ITI + prophylaxis with bypassing agents, and 33.3% prophylaxis; of these patients who received ITI at the time of the analysis, 12.5% reported ITI successful (negative inhibitors) and 87.5% were still in ITI.

A total of 50% of the participants (n = 6) were treated with plasma-derived coagulation factor VIII concentrate (pdFVIII), followed by 25% (n = 3) with emicizumab, 8.3% (n = 1) with aPCC, 8.3% (n = 1) with pdFVIII + aPCC, and 8.3% (n = 1) with pdFVIII + rFVIIa.

The most frequent mutation was an inversion of intron 22 (50%, n = 6), followed by a small duplication in exon 16 (16.67%, n = 2), with a non-null mutation in both cases, an inversion of intron 1 (8.33%, n = 1), a large deletion in exon 14 (8.33%, n = 1), a nonsense mutation of (8.33%, n = 1), and a splice site mutation (8.33%, n = 1). A total of 66.7% of patients had high inhibitor titers (Table 1).

A total of 83.3% of the patients presented null mutations; 16.67% (n = 2) presented a small duplication in exon 16 with a non-null mutation in both cases (Table 2).

We also analyzed the development of inhibitory antibodies, finding that 66.7% of the population (n = 8) developed high-titer inhibitory antibodies, of which five patients (62.5%) had an inversion of intron 22, one (12.5%) had an inversion of intron 1, one (12.5%) had a splice-site, and one (12.5%) had a small duplication (Table 3).

We did not find a statistically significant association between the type of mutation and the inhibitor titer, possibly due to the sample size; however, we found a trend that could associate the presence of high titers with the inversion of intron 22 (Table 3).

Regarding the association between null and non-null mutations (factor function) related to the type of mutation, we found a statistical significance with a *p* value of 0.03 between the null mutation and the inversion of introns 22, 1, 13 and exons 24 and 14. However, this association was affected by the frequency of intron 22 and its relation to the null mutation (Table 4).

This study shows the relation between the small duplication F8 mutation: c. [5447-5448dupGG]; (0) *p*. (Gln1817Glufs] with the non-null mutation; we found a significant association with a *p* value of 0.001. Of these two patients, one of them reported high inhibitor titers and the other one low inhibitor titers; therefore, it was correlated with factor traces, whose levels were below 0.1 in both patients. Reviewing the literature, no reports were found to associate this mutation with the risk of generating inhibitors.

## 4. Discussion

In our study, the inversion of intron 22 had a frequency of 50% (6/12), a finding that coincides with other studies, e.g., Garcés and Linares [11], with a frequency of 40% (12/30) in Colombian patients and Mantilla, Beltran et al. [12], with a frequency of 45% (14/31) in Mexican patients, for the same mutation. INV22 is estimated to be the most frequent in patients with hemophilia A, with a prevalence between 40% and 50% [6,13].

We found the inversion of intron 1 in 8.33% of the cases (1/12), coinciding with the finding of Garcés and Linares [11], of 10% (3/30), but different from the results obtained by Albánez and Ruiz et al. [14] and Mantilla and Beltran et al. [12], who did not report this mutation. It is important to note that intron 1 inversion appears in approximately 2% to 5% of severe cases of hemophilia [13,15].

Null mutations were found in 83.3% of patients and mutations at high risk of inhibitor development in 58.3% in the same population. As reported in the studies by Oldenburg and Pavlova et al. [6]. Gouw and van den Berg et al. [9], Rossetti and Szurkalo et al. [10] and the Institute of Experimental Medicine [3], there are mutations associated with the absence of a product gene (null mutation) and an increased risk of generating inhibitors, including an inversion of introns 1 and 22, large deletions, nonsense mutations and a splice site mutation.

According to the consideration of Spena and Garagiola et al. [16] regarding non-null mutations, defined as those that allow residual synthesis of the factor and that could play a protective role against the development of inhibitors, it was found in this study that 16.7% had non-null mutations and 83.3% had null mutations (n = 10), of which 70% had high titers.

One of the patients with low inhibitors included in our study had an intron 22 mutation. It has been observed that some patients with high-risk mutations do not develop inhibitors, so there must be other predisposing factors [3].

The small duplication F8: c. [5447-5448dupGG]; (0) p. (Gln1817Glufs] mutation of exon 16 had a frequency of 16.6%, showing a significant association with non-null mutations; however, reviewing the literature, no reports were found to associate this mutation with the risk of generating inhibitors and there were no reports of the mutation in the CDC listing with search date 4 June 2020.

## 5. Conclusions

Ten of the twelve patients (83.3%) presented null mutations (absence of factor), which are the most frequent in patients with hemophilia A with inhibitors. Inversion of introns 22 and 1, large deletions, nonsense mutations and splice site mutations were the determining mechanisms of these mutations.

Knowing the type of mutation and its association as a risk factor for generating inhibitors invites us to delve into other outcomes such as residual values of coagulation FVIII as well as its impact on the half-life of the exogenous factor applied in prophylaxis. This would allow individualized strategies to be proposed based on a genomic understanding.

## 6. Limitations

The sample size was too small, and statistical tests could not identify significant relationships within the study.

## Figures and Tables

**Table 1:** Description of variables.

Variable				Category	N	%
Treatment				ITI ITI-Prophylaxis Prophylaxis	6 2 4	50.0 16.7 33.3
Type of mutation				Inversion Intron 22 Small Duplication	6 2	50.0 16.7
				Inversion Intron 1 Splice site Nonsense Large Deletion	1 1 1 1	8.3 8.3 8.3 8.3
Inhibitor titers				High titers Low titers	8 4	66.7 33.3
Variable	Q1	Median	Q3	Minimum	Maximum	* *p* Value
Age (years)	7.0	13.50	20.75	6	48	0.019

* Shapiro–Wilk test.

**Table 2:** Inhibitor titers and mutations.

Inhibitors Titer (BU*)	NM_000132.3 (F8)-	Exon Intron	Null Mutation y Non Null
High titer (57BU)	Small duplication c. [5447-5448dupGG];(0) *p*. (Gln1817Glufs)	Exon 16	Non null
High titer (60 BU)	Inversion intron 22	Intron 22	Null
Low titer (4.8 BU)	Small duplication c. [5447-5448dupGG]; (0) *p*.(Gln1817Glufs)	Exon 16	Non null
High titer (7 BU)	Inversion intron 22	Intron 22	Null
Low titer (1.4 BU)	Inversion intron 22	Intron 22	Null
High titer (512 BU)	Inversion intron 1	Intron 1	Null
High titer (238 BU)	Inversion intron 22	Intron 22	Null
High titer (320 BU)	Splice site c. [2114-1G > C]; (0)	Intron 13	Null
Low titer (2.2 BU)	Nonsense mutation c. [6721C > T]; (0) *p*.(Gln2241Ter)	Exon 24	Null
High titer (48 BU)	Inversion intron 22	Intron 22	Null
High titer (8.0 BU)	Inversion intron 22	Intron 22	Null
Low titer (1.0 BU)	Large deletion	Exon 14	Null

* BU (Bethesda units).

**Table 3:** Association between type of mutation and presence or absence of inhibitors.

Mutation Type	High Titer	Low Titer	Total	*p* Value *
Inversion of intron 1 *F*8	1	0	1	0.46
Inversion of intron 22 *F*8	5	1	6	0.22
Large deletion of exon 14 *F*8	0	1	1	0.14
Mutation nonsense of exon 24 *F*8	0	1	1	0.14
Small duplication *F*8	1	1	2	0.58
Splice site *F*8	1	0	1	0.46
	8	4	12	

* Test Chi-squared.

**Table 4:** Association between type of mutation and null mutation.

Mutation Type	Null	Non Null	Total	*p* Value *
Inversion of intron 1 *F*8	1	0	1	0.035
Inversion of intron 22 *F*8	6	0	6	
Large deletion of exon 14 *F*8	1	0	1	
Nonsense mutation of exon 24 *F*8	1	0	1	
Small duplication *F*8	0	2	2	
Splice site *F*8	1	0	1	
	10	2	12	

* Test Chi-squared.

## Data Availability

The data presented in this study are available on request from the corresponding author, due to restrictions for privacy and ethical. The data are not publicly available due to it is data of patients and belong to a health institution in Colombia.
